# Construction, operations, and sustainability of maternal and child health pharmacy clinics in China: a national multicenter cross-sectional survey

**DOI:** 10.1186/s12913-026-14586-z

**Published:** 2026-04-22

**Authors:** Yifan Li, Ran Wang, Xin Yu, Xin Feng

**Affiliations:** Department of Pharmacy, Beijing Obstetrics and Gynecology Hospital, Beijing Maternal and Child Health Care Hospital, Capital Medical University, Beijing, 100026 China

**Keywords:** Maternal and child health, Pharmacist-managed clinics, Pharmaceutical care, Financial sustainability, Health policy, Surveys and questionnaires, China

## Abstract

**Background:**

Pharmacist-managed clinics are being promoted nationally in China to strengthen pharmaceutical care, particularly maternal and child health (MCH). However, little is known about how these clinics are structured, staffed, financed, or sustained at a national level. This study examined the development, operational characteristics, and financial viability of MCH pharmacist-managed clinics in China.

**Methods:**

A national, multi-center, cross-sectional survey was conducted from August to September 2025 among pharmacy department leaders from 150 medical institutions across 32 provinces. A validated 44-item questionnaire assessed institutional characteristics, clinic establishment, service delivery models, workforce composition, financial status, and operational challenges (5-point Likert scales). Descriptive statistics were used to summarize the clinical characteristics. Multivariable logistic regression identified factors associated with financial sustainability, defined as achieving a break-even or profitability.

**Results:**

Ninety valid responses were included (response rate: 62.7%). Among the participating institutions, 75/90 (83.3%) had established pharmacist-managed clinics, and 52/90 (57.8%) operated dedicated MCH clinics. Overall, 58/90 (64.4%) institutions provided services free of charge, and 55/90 (61.1%) reported operating at a financial deficit. Only 30/90 (33.3%) institutions charged fees for pharmacy consultations, and only 3/90 (3.3%) reported profitability. Annual patient volumes varied substantially among the 52 MCH clinics (median 142; IQR 27.5–345.8), with nearly half of the MCH clinics (25/52, 48.1%) serving fewer than 100 patients per year. The most reported barriers were low patient willingness to pay (mean score 4.1 ± 1.1 on a 5-point Likert scale) and the absence of unified national charging standards (4.1 ± 1.0). In adjusted analyses, service charging was the strongest predictor of financial sustainability (aOR = 3.85; 95% CI 1.88–7.89), followed by the inclusion of clinic activity in performance-based compensation (aOR = 2.15; 95% CI 1.10–4.20). Institutional characteristics, duration of service provision, and pharmacists’ seniority were not independently associated with sustainability.

**Conclusions:**

MCH pharmacist-managed clinics have expanded rapidly nationwide, but the structural misalignment between policy-driven adoption and financial and workforce support has created significant sustainability challenges. Long-term viability requires coordinated reforms, including standardized fee schedules, integration of pharmacist services into insurance reimbursement, and strengthened institutional investment in the workforce and service pathways. These changes are essential to ensure that MCH pharmacy clinics can sustainably improve medication safety and health outcomes for mothers and children.

**Supplementary Information:**

The online version contains supplementary material available at 10.1186/s12913-026-14586-z.

## Background

Pharmacist-managed clinics represent an important evolution in pharmacy practice, shifting from product-oriented dispensing to patient-centered clinical care. Evidence demonstrates that such clinics improve medication safety, enhance adherence, and reduce healthcare costs by delivering structured, expert-led pharmaceutical services [1]. Specialized medication management is particularly important in maternal and child health (MCH). Pregnant, lactating, and pediatric patients frequently require pharmacotherapy and are often excluded from clinical trials, placing them at an elevated risk of adverse drug events and inappropriate medication use [2, 3].

In China, national health system reforms, particularly the Healthy China 2030 strategy and the 2020 directive on rational medication use, have accelerated the establishment of pharmacist-managed clinics within hospitals. Many obstetrics, gynecology, and pediatric departments have subsequently developed MCH-focused pharmacy clinics to improve medication counseling and support safe prescribing [4–6]. However, the rapid expansion of these services has outpaced the development of standardized operational models, sustainable financing mechanisms, charging policies, and workforce requirements.

Although the number of clinics continues to grow, no national-level health service evaluation has examined how these clinics are structured, staffed, funded, or integrated into routine care. Existing evidence is limited to single-institution reports that do not assess broader system issues, including financial sustainability, reimbursement pathways, performance incentives, patient demand, and regional variation [7, 8]. This gap limits policymakers and hospital leaders’ ability to design evidence-based strategies for service planning, resource allocation, and long-term sustainability.

Given the rapid but uneven development of MCH pharmacist-managed clinics, a comprehensive national assessment is needed to characterize their current status and identify the organizational and financial challenges affecting their implementation in China’s health system.

## Methods

### Study aim, design, and setting

This study aimed to evaluate the construction, operational characteristics, and sustainability of maternal and child health (MCH) pharmacist-managed clinics in China. We conducted a national, multi-center, cross-sectional survey from August 1 to September 1, 2025, following the STROBE reporting guidelines for observational studies. The study was conducted in accordance with the Declaration of Helsinki. Ethical approval was obtained from the Ethics Committee of Beijing Obstetrics and Gynecology Hospital, Capital Medical University (Approval No. 2024-KY-035-01; Approval date Apr 11, 2024). Electronic informed consent was obtained from all respondents prior to questionnaire completion.

### Participants and sampling frame

Directors of the pharmacy department were recruited from 150 medical institutions affiliated with the Women and Children Specialized Pharmacists Branch of the Chinese Pharmacists Association. Eligible respondents were required to (1) have administrative responsibility for pharmacy services, and (2) possess direct knowledge of their institution’s pharmacist-managed clinical operations. Directors from institutions without clinics were included to capture the perceived barriers. Questionnaires with more than 20% of missing data were excluded.

### Questionnaire development and validation

This study employed a self-designed questionnaire (supplementary file 1). Data were collected using a structured 44-item questionnaire developed through (1) expert consultation involving five senior specialists in clinical pharmacy and MCH services and (2) review of national policy documents, relevant literature, and established frameworks for pharmacy services.

Psychometric testing demonstrated strong reliability (Cronbach’s α = 0.87) and content validity (CVI α = 0.91). Questionnaire domains included: (1) institutional characteristics: hospital level, ownership, region, and bed capacity; (2) clinic infrastructure and workforce: year of establishment, physical space, number, and qualifications of pharmacists; (3) service delivery and operations: patient volume, service types, and charging policies; and (4) sustainability and challenges, assessed via 5-point Likert scales (1 = not important; 5 = extremely important).

A pilot test of eight institutions was conducted to evaluate clarity and feasibility.

### Data collection procedures

The survey was conducted using a secure online platform. The respondents were able to review and revise the entries before submission. All responses were screened for completeness. Minor missing data (< 5%) were managed with pairwise deletion for descriptive statistics, whereas questionnaires with major missing data (> 20%) were excluded. Financial sustainability was defined as operating at break-even or generating positive net revenue, as reported by each institution.

### Statistical analysis

The data analysis was performed using SPSS version 26.0 (IBM Corp., Armonk, NY, USA). Normality was assessed using the Shapiro–Wilk test. Continuous variables are presented as means ± standard deviations (SD) or medians with interquartile ranges (IQR) and compared using t-tests, ANOVA, or Mann–Whitney U tests, as appropriate. Categorical variables were summarized as frequencies and percentages and were analyzed using Chi-square or Fisher’s exact tests. No sampling weights or regional stratification adjustments were applied, as the survey sought to describe observed patterns among participating institutions rather than produce population-representative estimates. For exploratory comparisons, clinics were categorized by establishment year (≤ 2022 vs. > 2022) to examine whether earlier implementation was associated with higher service volumes or financial performance.

To identify the independent predictors of financial sustainability (defined as break-even or profitability), a multivariable logistic regression model was constructed. Candidate variables were selected based on their theoretical relevance and univariate significance (*P* < 0.10). Multicollinearity was assessed using variance inflation factors (VIF < 5), and model fit was evaluated using the Hosmer–Lemeshow test. All statistical tests were two-tailed, and significance was defined as *P* < 0.05.

## Results

### Response rate

A total of 94 questionnaires were received from 150 invited institutions (response rate: 62.7%). After excluding incomplete responses, 90 valid questionnaires were included (effective response rate: 95.7%).

### Institutional characteristics

These institutions represented all 32 provinces in China (Table 1: Characteristics of participating institutions). Most of them were tertiary-level public hospitals (90%). Both institutional capacity and pharmacy workforce composition varied widely, with bed numbers and staffing levels ranging from small local centers to large academic hospitals. This heterogeneity reflects substantial differences in service scale, organizational resources, and maturity of pharmacy services across regions.

**Table 1:** Characteristics of participating institutions (*n* = 90)

Characteristic	Category / Statistic	*n* (%) / Median (IQR)
Geographic distribution	Eastern region	36 (40.0%)
	Central region	21 (23.3%)
	Western region	33 (36.7%)
Institution type	Tertiary specialized hospital	48 (53.3%)
	Tertiary general hospital	33 (36.7%)
	Secondary specialized hospital	7 (7.8%)
	Secondary general hospital	1 (1.1%)
	Other facility (local medical center)	1 (1.1%)
Ownership	Public institution	88 (97.8%)
	Private for-profit institution	2 (2.2%)
Hospital capacity	Bed capacity, median (IQR)	800 (400–1496)
Pharmacy personnel	Total pharmacy professionals, median (IQR)	47.5 (24.3–80.8)
Educational background	Doctoral degree holders, median (IQR)	0 (0–2)
	Master’s degree holders, median (IQR)	10 (2–21.8)
Professional title	Senior pharmacist, median (IQR)	1 (0–3)
	Deputy senior pharmacist, median (IQR)	4.5 (2–10.8)
	Intermediate pharmacist, median (IQR)	20 (9.3–34.8)
Clinical pharmacist certification	National certification, median (IQR)	6.5 (3.3–10)
	Provincial certification, median (IQR)	0 (0–0.8)

Note: Data are presented as n (%) or median (interquartile range, IQR) unless otherwise specified

### Pharmacy clinic establishment

Among the 90 institutions, 75/90 (83.3%) had established pharmacist-managed clinics and 52/90 (57.8%) operated dedicated MCH specialty clinics (Table 2: Establishment and operational characteristics of pharmacy clinics). Insufficient pharmacist staffing and shortage of qualified specialty practitioners were the most cited barriers to clinical establishment.

Charging practices varied considerably. Only a minority of clinics were charged for services 30/90 (33.3%). Among institutions that charged fees, consultation prices were modest, generally ranging from ¥15 to ¥30 depending on pharmacist seniority. Most clinics reported operating at a financial deficit 55/90 (61.1%). More than half of institutions 48/90 (53.3%) lacked performance incentive mechanisms linked to clinic activity, indicating limited integration of pharmacy clinics into institutional financial and human resource systems.

**Table 2:** Establishment and operational characteristics of pharmacy clinics (*n* = 90)

Characteristic	Category / Description	*n* (%) / Median (IQR)
Pharmacy clinic establishment	Institutions with established pharmacy clinics	75 (83.3%)
	Institutions without pharmacy clinics	15 (16.7%)
Maternal and child specialty clinics	Institutions with independent clinics and fixed scheduling	52 (57.8%)
Motivations for establishing clinics	Response to national policy initiatives	61 (67.8%)
	Enhancement of pharmacy discipline influence	58 (64.4%)
	Meeting specific patient needs	57 (63.3%)
	Improvement of medical service quality	54 (60.0%)
Barriers among institutions without clinics (*n* = 15)	Insufficient pharmacist workforce	11 (73.3%)
	Lack of qualified specialty pharmacists	9 (60.0%)
	Absence of clear service charging standards	6 (40.0%)
	Low patient demand or willingness to pay	6 (40.0%)
Service fee structure	All services provided free of charge	58 (64.4%)
	All services charged	30 (33.3%)
	Selective charging	2 (2.2%)
Basis for fee determination (charging institutions, *n* = 32)	Equivalent to physician consultation fees	15 (46.9%)
	Provincial/municipal health insurance regulations	12 (37.5%)
	Hospital-determined pricing	5 (15.6%)
Consultation fee standards	Senior pharmacists, median (IQR)	20.5 (13.75–30)
	Deputy senior pharmacists, median (IQR)	20 (11.6–30)
	Intermediate pharmacists, median (IQR)	15 (8–26.25)
Financial sustainability	Deficit operation	55 (61.1%)
	Break-even	32 (35.6%)
	Profitable	3 (3.3%)

Note: Data are presented as n (%) or median (interquartile range, IQR) unless otherwise specified

*Motivations* and *barriers* allow multiple selections

### Clinic operations and service provision

Across the 52 MCH clinics, the service types varied, with pregnancy medication consultation 9/52 (17.3%), pediatric medication guidance 6/52 (11.5%), and lactation-related counseling 5/52 (9.6%) being the most common (Table 3: Characteristics of maternal and child specialty pharmacy clinics). The annual patient volume showed substantial variability (median 142; IQR 27.5–345.8). Nearly half of the clinics served fewer than 100 patients per year, whereas a small subset managed very high volumes (> 700 annually), highlighting significant differences in service uptake and clinic maturity across institutions and regions.

Most clinics operate a limited number of weekly sessions and are staffed by small teams, typically three dedicated pharmacists, often with advanced degrees or senior titles. However, formal specialty certification remains uncommon.

**Table 3:** Characteristics of maternal and child specialty pharmacy clinics (*n* = 52)

Characteristic	Category / Description	*n* (%) / Median (IQR)
Clinic service types	Pregnancy medication consultation	9 (17.3%)
	Pediatric medication guidance	6 (11.5%)
	Lactation/pregnancy medication services	5 (9.6%)
Annual patient service volume	Median (IQR)	142 (27.5–345.8)
	0–100 patients	25 (48.1%)
	101–300 patients	13 (25.0%)
	> 700 patients	7 (13.5%)
Clinic operation schedule	Weekly clinic sessions (half-day units), median (IQR)	2 (1–5)
	Planned patient capacity per half-day session per pharmacist, median (IQR)	3 (1–5)
Pharmacy clinic staffing	Fixed clinic pharmacists, median (IQR)	3 (2–7)
	With deputy senior titles or above, median (IQR)	2 (1–3)
	With master’s degree or above, median (IQR)	2 (1–3)
	Clinical experience (years), median (IQR)	10 (6.75–12.25)
Specialty certification	Obstetrics/gynecology specialty pharmacist, median (IQR)	1 (0–1)
	Pediatric specialty pharmacist, median (IQR)	1 (0–1)

Note: Data are presented as n (%) or median (interquartile range, IQR) unless otherwise specified

### Service content and quality assurance

The most frequently consulted topics included adverse drug reactions, medication selection, dosing, drug interactions, and pregnancy/lactation-related medication safety. Commonly inquired medications were levofloxacin, ibuprofen, ribavirin, loratadine, and acetaminophen. Evidence-based resources were widely used, including international or national guidelines (85.6%), drug package inserts (83.3%), pharmacy databases (71.1%), and clinical research literature (32.2%). Standardized documentation practices varied across institutions: 32/90(35.6%) provided structured forms for all first-visit patients, 24/90(26.7%) used them selectively, and 20/90(22.2%) did not use them routinely. Patient satisfaction assessments were conducted regularly in 9/90(10.0%) of clinics and irregularly in 23/90(25.6%), with reported satisfaction levels generally high.

### Challenges and barriers

The institutions reported challenges across the four dimensions (Table 4: Challenges in specialty pharmacy clinic development assessed across three dimensions). External recognition and policy constraints, particularly the absence of unified charging standards and limited patient willingness to pay, received the highest severity scores, representing the most prominent systemic barriers to clinical development. Talent development issues and internal operational pressures, including heavy workloads and lack of performance-based incentives, were also consistently rated as significant challenges. In contrast, challenges related to service standardization scored moderately, indicating partial progress in workflow implementation but ongoing gaps in follow-up processes and continuity of care.

**Table 4:** Challenges in specialty pharmacy clinic development assessed across three dimensions (5-point Likert scale, *n* = 90)

Dimension	Item	Mean ± SD
Service standardization	Patient reception and consultation workflow	3.3 ± 1.5
	Standardized documentation and record management	3.2 ± 1.3
	Patient follow-up and continuity of care	2.9 ± 1.4
Talent development	Lack of systematic pharmacist training programs	3.7 ± 1.2
	Insufficient clinical practice skills among pharmacists	3.7 ± 1.1
	Limited career advancement pathways in clinical pharmacy	3.6 ± 1.1
External recognition and policy environment	Lack of unified national charging standards	4.1 ± 1.0
	Low patient awareness and willingness to pay	4.1 ± 1.1
	Inconsistent policy or regulatory support	3.9 ± 1.1
Internal operational and institutional support	Excessive workload	3.9 ± 1.1
	Lack of performance-based incentive mechanisms	4.0 ± 1.0
	Insufficient research funding and institutional support	4.0 ± 1.0

Note: Ratings were based on a 5-point Likert scale (1 = not challenging, 5 = extremely challenging). Data are expressed as mean ± standard deviation (SD)

### Factors associated with clinic establishment and sustainability

The clinic establishment rates did not differ significantly by hospital level (*P* = 0.106). Among the established clinics, those launched before 2022 had higher patient volumes and were more likely to charge for services (*P* < 0.01). Charging practices were strongly associated with financial sustainability, with clinics that charged fees being significantly more likely to achieve break-even or profitability (*P* = 0.002; Table 5: Univariate analysis of factors associated with pharmacy MCH specialty clinic and operational outcomes).

In multivariable logistic regression, service charging remained the strongest independent predictor of financial sustainability (adjusted odds ratio [aOR] = 3.85; 95% CI: 1.88–7.89), and the inclusion of clinic activity in performance-based compensation was also a significant predictor (aOR = 2.15; 95% CI: 1.10–4.20). Structural institutional characteristics such as hospital level, clinic duration, and pharmacist seniority were not associated with sustainability, suggesting that financial and incentive mechanisms play a more determinant role than organizational scale or staffing profiles.

**Table 5:** Univariate analysis of factors associated with pharmacy MCH specialty clinic and operational outcomes

Analysis / Variable	Group 1	Group 2	*P*-value
Part A: Clinic establishment by hospital level			
Hospital level	Tertiary (*n* = 81)	Secondary (*n* = 8)	0.106ᵃ
Institutions with established clinic, n/N (%)	49/81 (60.5%)	3/8 (37.5%)	—
Part B: Operational outcomes among established clinics			
Annual patient volume by establishment year	Established ≤ 2022 (*n* = 16)	Established > 2022 (*n* = 32)	0.003ᵇ
Median (IQR)	433 (195–1072)	112 (50–291)	—
Charging rate by establishment year	Established ≤ 2022 (*n* = 16)	Established > 2022 (*n* = 32)	0.005ᵃ
Institutions charging for services, n/N (%)	14/16 (87.5%)	13/32 (40.6%)	—
Financial sustainability by charging policy	Charging clinics (*n* = 27)	Non-charging clinics (*n* = 25)	0.002ᵃ
Achieving break-even or profitability, n/N (%)	17/27 (63.0%)	4/25 (16.0%)	—

Note: Boldface indicates statistical significance (*P* < 0.05)

ᵃ Chi-square test or Fisher’s exact test

ᵇ Mann–Whitney U test

Some subgroup counts may not total *n* = 75 due to missing data

## Discussion

This national multicenter survey provides the first comprehensive examination of maternal and child health pharmacist-managed clinics across China, revealing a striking disconnect between rapid institutional adoption and fragile financial sustainability. While 83.3% of participating institutions had established pharmacist-managed clinics and 57.8% operated dedicated MCH specialty clinics, the operational reality was considerably less encouraging: 61.1% reported financial deficits, annual patient volumes varied substantially, and nearly half of MCH clinics served fewer than 100 patients per year. Multivariable analysis identified service charging and integration of clinic activity into performance-based compensation as the principal determinants of financial sustainability, whereas structural institutional factors—including hospital level, clinic duration, and pharmacist seniority—were not independently associated with this outcome. These findings indicate that the sustainability challenge is fundamentally rooted in system-level financing and incentive design rather than in clinical capability or institutional scale.

The clinic establishment rate observed in this study exceeds previous national estimates for general hospital pharmacy services [9], likely reflecting both the targeted policy impetus within the MCH sector and the composition of our sample, which was drawn from institutions affiliated with a national professional association and may therefore overrepresent early adopters. Nevertheless, the pattern of high adoption accompanied by low utilization and financial fragility aligns with international experience. In the U.S., pharmacist-led services in chronic disease management and primary care have demonstrated clinical effectiveness and cost-effectiveness; however, their scalability remains constrained by inconsistent reimbursement pathways, limited billing mechanisms, and productivity metrics that inadequately capture cognitive pharmacy services [10–12]. In England, the volume and integration of community pharmacy services are strongly influenced by commissioning structures and payment models, reinforcing how remuneration frameworks directly shape service delivery [13]. Even in specialized domains, such as palliative care, challenges persist in defining, integrating, and funding pharmacist contributions within existing payment structures [14]. These international parallels highlight that the sustainability challenges identified in our study are not unique to China but reflect a broader structural misalignment—observed across diverse health systems—between the recognized clinical value of pharmacist interventions and the financial mechanisms available to support them [15, 16].

Several systemic and behavioral mechanisms may explain this misalignment. At the structural level, pharmaceutical care in China lacks standardized billing codes and reimbursement pathways, fundamentally limiting cost recovery and reducing institutional incentives for sustained investment [17]. The absence of unified national charging standards, identified in our survey as the highest-rated barrier, creates an environment in which individual institutions must absorb the full cost of service provision without regulatory support for revenue generation. At the organizational level, the finding that 53.3% of institutions lacked performance incentive mechanisms linked to clinic activity suggests that pharmacy clinics remain peripheral to institutional management systems, consistent with earlier evaluations of pharmacy service models in China [18]. Behavioral economics offers additional explanatory insights: both administrators and patients may exhibit present bias, systematically undervaluing services whose benefits—such as reduced adverse drug events or improved medication adherence—accrue over the medium-to long-term rather than immediately [19]. For patients, this may manifest as a low willingness to pay for preventive counseling; for administrators, it may reduce the priority accorded to resource allocation for pharmacy clinics. Moreover, default bias likely contributes to underutilization, as pharmacy consultations are not embedded as a routine step in maternal care pathways, requiring additional effort from both patients and care teams to initiate engagement. These interconnected mechanisms suggest that the observed challenges are deeply rooted in system design and incentive architecture, rather than reflecting deficiencies in clinical competence or individual motivation.

The implications for policy and practice are clear and urgent. At the national level, establishing standardized charging codes for pharmacist consultations and integrating these services into medical insurance reimbursement would directly address the most prominent barrier identified in this study. A substantial body of international evidence supports the economic value of pharmacist-led care in chronic disease management and maternal health, including documented reductions in adverse drug events and downstream healthcare utilization [20]. At the institutional level, hospitals should consider embedding pharmacist consultations as a default component of maternity care pathways—an approach supported by evidence from other preventive health interventions and consistent with behavioral strategies to overcome the utilization barriers identified here. Linking clinical productivity to performance-based compensation could further enhance pharmacist engagement and promote service quality. The expansion of accredited training pathways and specialty certification programs in obstetrics, gynecology, and pediatrics would also strengthen workforce capacity and help address the personnel shortages that remain the most frequently cited barrier among institutions without established clinics.

This study has several limitations. Our sample was recruited through a professional association network, which likely resulted in overrepresentation of institutions already engaged in specialty pharmacy practice; the clinic establishment rate of 83.3% exceeds previous national estimates [6], suggesting participation bias toward early adopters. Cross-sectional design captures a single point in time and cannot establish causal relationships between organizational factors and sustainability outcomes. Operational and financial data were self-reported and may be subject to recall or social desirability bias. The findings predominantly reflect tertiary public hospitals and may not be generalizable to lower-level or private institutions. Finally, formal cost effectiveness or budget impact analyses were beyond the scope of this study but are essential for informing evidence-based reimbursement policies. Future research should employ stratified national sampling to enhance representativeness, longitudinal designs to assess the impact of policy changes over time, and rigorous economic evaluations to quantify the return on investment for MCH pharmacist-managed services. Intervention studies testing behavioral strategies, such as default scheduling or structured follow-up protocols, may also yield insights into improving clinical utilization and long-term sustainability.

## Conclusion

This national assessment shows that MCH pharmacist-managed clinics have expanded rapidly in response to policy initiatives, yet their development remains structurally misaligned with the financial and workforce support required for sustainable operations. Most clinics face persistent deficits, low patient volumes, and shortages of qualified personnel, reflecting systemic gaps rather than clinical shortcomings. To ensure long-term viability, national health authorities should establish unified charging standards and incorporate pharmacist consultations into insurance reimbursements. At the institutional level, hospitals must strengthen their workforce development and integrate pharmacy services into routine care pathways. Coordinated policy and organizational action are essential to transforming these clinics from policy-driven pilots into sustainable components of maternal and child health services.

## Electronic Supplementary Material

Below is the link to the electronic supplementary material.

Supplementary Material 1

## Data Availability

The datasets generated and analyzed during the current study are not publicly available because of institutional confidentiality and restrictions related to identifiable organizational information but are available from the corresponding author upon reasonable request.
