# Supplementary material for: Construction, operations, and sustainability of maternal and child health pharmacy clinics in China: a national multicenter cross-sectional survey
Source: BMC Health Serv Res. 2026 Apr 22;26:796. doi: 10.1186/s12913-026-14586-z (PMC13244887; doi:10.1186/s12913-026-14586-z)
Supplement: Supplementary file 1 — Supplementary Material 1 [file 12913_2026_14586_MOESM1_ESM.docx]

**Appendix: Survey Instrument (English Translation)**

**Survey on the Establishment, Operation, and Development of Women and Children’s Specialty Pharmacy Outpatient Clinics**

Dear Head of Pharmacy Department / Head of Pharmacy Outpatient Clinic:

Greetings!

To implement the national strategic initiatives for enhancing the high-quality development of pharmacy services and to accurately understand the development trajectory and practical needs of women-and-children specialty pharmacy outpatient clinics in China, the Women and Children Pharmacists Branch of the Chinese Pharmaceutical Association is conducting this in-depth survey.

This survey aims to comprehensively and systematically collect key information on the establishment foundation, operational models, service scope, quality management, challenges, and expectations for development of women-and-children specialty pharmacy outpatient clinics (particularly services related to medication use during pregnancy and lactation, pediatric medication, and reproductive medication, etc.). Your valuable data and insights will provide critical empirical evidence for depicting the current landscape, revealing regional and institutional differences, analyzing influencing factors, summarizing successful experience, formulating development strategies, and promoting policy improvement, thereby jointly advancing women-and-children pharmacy services to a new level.

This questionnaire is completed anonymously. All data will be used only for statistical analysis and the drafting of an industry report, and we will strictly adhere to confidentiality principles. The questionnaire is relatively detailed; thank you for your patience and careful completion.

Thank you again for taking your valuable time to participate in this in-depth survey.

Contact: Yifan Li +86-10-5227 7242

# Part I. Institutional Demographics

1. Full name of institution: ___________________________ [Fill-in] *

2. Province/Autonomous Region/Municipality: ___________ [Fill-in] *

3. City/District/County: ______________ [Fill-in] *

4. Institution level: [Single choice] *
○ Tertiary general hospital
○ Tertiary specialty hospital
○ Secondary general hospital
○ Secondary specialty hospital
○ Other (please specify): _________________ *

5. Institution type: [Single choice] *
○ Public
○ Non-profit private
○ For-profit private

6. Number of licensed/open beds (as of December 31, 2024): _____ beds [Numeric entry] *

7. Pharmacy professional staff (as of December 31, 2024) [Numeric entry]
Total pharmacy professionals: _____ persons. Among them,
PhD pharmacists: _____;
Master’s degree pharmacists: _____;
Chief/senior-title pharmacists: _____;
Associate chief/senior-title pharmacists: _____;
Supervisory-title pharmacists: _____;
Holders of National Clinical Pharmacist Training Certificate: _____;
Holders of Provincial Clinical Pharmacist Training Certificate: _____
[Fill-in] *

8. Pharmacy department research output (2022–2024) [Numeric entry]
SCI/SSCI-indexed publications: _____ papers;
Publications in Chinese core journals: _____ papers;
Guidelines/expert consensus authored: _____ documents;
Total funding for national-level projects led: approx. _____ (10,000 CNY);
Total funding for provincial/ministerial projects led: approx. _____ (10,000 CNY)
[Fill-in] *

# Part II. Clinic Establishment & Operation

9. Has your institution established a pharmacy outpatient clinic? [Single choice] *
○ Yes
○ No

10. If your institution has NOT established a specialty pharmacy outpatient clinic, what are the main barriers? (Select up to 3) [Multiple choice] *
□ Lack of competent specialty pharmacists
□ Severe shortage of pharmacist workforce
□ Lack of clear pharmacy service charging standards
□ Insufficient support from hospital leadership/management
□ Low awareness of clinical departments / low willingness to refer
□ Estimated low patient demand and/or willingness to pay
□ Lack of facilities/IT systems and other hardware support
□ Other (please specify): _________________
(Displayed if Q9 = No)

11. Does your institution plan to establish a specialty pharmacy outpatient clinic within the next 1–3 years? [Single choice] *
○ Yes, clear plan; preparation is underway
○ Preliminary intention; feasibility assessment ongoing
○ No plan at present
(Displayed if Q9 = No)

12. Does the women-and-children specialty pharmacy outpatient clinic operate independently with a fixed schedule? [Single choice] *
○ Yes
○ No

13. Year the first women-and-children specialty pharmacy outpatient clinic was established: _____ [Numeric entry]
How many independent women-and-children specialty pharmacy outpatient clinics are operated? (e.g., pregnancy medication clinic and pediatric medication clinic each counts as 1): _____ [Numeric entry]
Which independent women-and-children specialty pharmacy outpatient clinics are currently operated? _____ (e.g., pregnancy medication clinic, etc.)
[Fill-in] *

14. Clinic workload (please provide annual data for 2024) [Numeric entry] *
Annual total service visits: _____ visits
Average weekly clinic sessions (half-day counts as 1 session): _____ sessions/week
Planned number of patients per pharmacist per clinic session (half-day): _____ patients
[Fill-in] *

15. Composition of outpatient pharmacist team (as of Dec 31, 2024) [Numeric entry] *
Total fixed outpatient pharmacists: _____
Associate senior title or above: _____
Master’s degree or above: _____
Average years of practice: approx. _____ years
National/provincial obstetrics & gynecology specialty clinical pharmacist certificate: _____
National/provincial pediatrics specialty clinical pharmacist certificate: _____
[Fill-in] *

16. When your institution decided to establish women-and-children specialty pharmacy outpatient clinics, what were the most important driving factors? (Select 1–3) [Multiple choice] *
□ Responding to national policies
□ Improving quality of medical services
□ Meeting specific patient needs
□ Enhancing the influence of the pharmacy discipline
□ Recommendations/needs from clinical departments
□ Hospital strategic development plan
□ Learning from successful experience of other hospitals
□ Pharmacy department’s own development initiative
□ Other: _________________

17. Is the maternal and child specialty pharmacy clinic a fee-based service? [Single choice] *
○ Yes, all specialty directions are charged
○ Yes, only some directions are charged (please specify): _________________
○ No, none are charged

18. Primary basis for the charging standard: [Single choice] *
○ Refer to the consultation fee of physicians at the same professional level in this hospital
○ Pharmacy service fee standards issued by provincial/municipal medical insurance authority or health commission (please specify the document): _________________
○ Hospital-set pricing (filed with the pricing authority)
○ Other (please specify): _________________ *

19. [If charged] Please specify the fee (CNY/visit). If not differentiated, enter the same value: [Numeric entry] *
* Chief/senior-title pharmacist: _____ CNY/visit
* Associate chief/senior-title pharmacist: _____ CNY/visit
* Intermediate and below: _____ CNY/visit [Fill-in] *

20. Compared with the period when services were free of charge, what impact do you think charging has on visit volume? [Single choice] *
○ Significant increase (>20%)
○ Slight increase (5%–20%)
○ No substantial impact (<5%)
○ Slight decrease (5%–20%)
○ Significant decrease (>20%)
○ No comparable free-of-charge period / difficult to judge

21. At the hospital level, is the service output of the pharmacy outpatient clinic included in pharmacists’ performance-based pay distribution? [Single choice] *
○ Yes, directly reflected
○ Somewhat indirectly reflected
○ Not reflected at all

22. Financial balance of women-and-children specialty pharmacy outpatient clinics: [Single choice] *
○ Profitable
○ Break-even
○ Loss-making

# Part III. Service Content & Quality Control

23. In your women-and-children specialty pharmacy outpatient clinics, what are the top 10 most frequently consulted categories of questions from patients? (Rank by frequency; 1 = most frequent) (e.g., drug selection, dosage and administration, adverse reactions, medication safety during pregnancy/lactation, drug interactions, etc.)
(1) ___________________________
(2) ___________________________
(3) ___________________________
(4) ___________________________
(5) ___________________________
(6) ___________________________
(7) ___________________________
(8) ___________________________
(9) ___________________________
(10) __________________________
[Fill-in] *

24. In your women-and-children specialty pharmacy outpatient clinics, what are the top 10 most frequently involved medicines (generic names)? (Rank by frequency; 1 = most frequent)
(1) ___________________________
(2) ___________________________
(3) ___________________________
(4) ___________________________
(5) ___________________________
(6) ___________________________
(7) ___________________________
(8) ___________________________
(9) ___________________________
(10) __________________________
[Fill-in] *

25. Main services provided (please check all that apply) [Multiple choice] *
□ Medication assessment and consultation during pregnancy
□ Medication assessment and consultation during lactation
□ Medication guidance for common pediatric diseases
□ Medication management for chronic pediatric diseases (e.g., asthma, epilepsy)
□ Consultation on medications related to reproductive endocrinology
□ Interpretation and consultation of pharmacogenetic testing
□ Other (please specify): __________ _________________

26. When providing services, what are the main evidence sources referenced by attending pharmacists? (Select no more than 3) [Multiple choice] *
□ Authoritative domestic and international clinical guidelines (e.g., Chinese Medical Association, ACOG, AAP)
□ Drug package inserts/labels (domestic and originator products)
□ Professional drug information databases (e.g., Micromedex, Lexicomp, UpToDate, AHFS-DI)
□ Pharmacotherapy monographs/textbooks
□ High-quality clinical research literature (RCTs, meta-analyses)
□ Hospital internal medication protocols / experience summaries
□ Pharmacist’s personal clinical experience
□ Other (please specify): _________________ *

27. Do your clinics provide standardized written pharmaceutical care records or medication recommendations (i.e., an “outpatient medication record”)? [Single choice] *
○ Yes. Provided to every first-time patient; provided for follow-ups as needed
○ Yes. Provided selectively based on complexity or patient needs
○ No. Only brief instruction sheet or verbal advice; no complete record
○ No. Written materials are not routinely provided

28. What contents are typically included in the outpatient medication record? (Multiple choice) *
□ Patient demographics and chief complaint
□ History of present illness, past history, medication history, allergy history
□ Pharmaceutical assessment (identification of drug-therapy-related problems)
□ Pharmacist interventions and recommendations (therapy adjustment, dose/administration, precautions)
□ Key points of medication education
□ Follow-up plan
□ Other (please specify): _________________ *

29. Does your institution conduct patient satisfaction surveys for the clinic services? [Single choice] *
○ Yes, regularly (e.g., quarterly/semiannually/annually)
○ Yes, irregularly
○ No, not conducted systematically yet

30. Approximate overall patient satisfaction in 2024: [Single choice] *
○ Very satisfied (≥95% satisfied/very satisfied)
○ Relatively satisfied (85%–94% satisfied/very satisfied)
○ Moderate (70%–84% satisfied/very satisfied)
○ Less satisfied (<70% satisfied/very satisfied)
○ No formal statistics / data unavailable

31. Please rate the standardization level of each core service process (1 = no unified standard, practice mainly relies on individual pharmacists’ experience; 5 = clear SOPs and strictly implemented) [Matrix] *

|  | 1 | 2 | 3 | 4 | 5 |
| --- | --- | --- | --- | --- | --- |
| Patient intake and information collection | ○ | ○ | ○ | ○ | ○ |
| Pharmaceutical assessment and problem identification | ○ | ○ | ○ | ○ | ○ |
| Intervention/recommendation and communication | ○ | ○ | ○ | ○ | ○ |
| Medication education | ○ | ○ | ○ | ○ | ○ |
| Outpatient medication record documentation | ○ | ○ | ○ | ○ | ○ |
| Patient follow-up | ○ | ○ | ○ | ○ | ○ |

32. Approximately how many sessions/days of women-and-children specialty pharmacy-related training are organized or funded each year? [Fill-in] *
_________________________________

33. Approximate average annual training budget per outpatient pharmacist: [Fill-in] *
_________________________________

34. Approximate annual investment for IT system development and maintenance related to pharmacy outpatient clinics: [Fill-in] *
_________________________________

35. What percentage of total weekly working time is spent on outpatient clinic work (including direct service and preparation)? [Fill-in] *
_________________________________

# Part IV. Challenges & Future Needs

36. In terms of workforce development and professional competency improvement, what are the main challenges? (Rate importance: 1 = Not important at all; 2 = Slightly important; 3 = Moderately important; 4 = Very important; 5 = Extremely important) [Matrix] *

|  | 1 | 2 | 3 | 4 | 5 |
| --- | --- | --- | --- | --- | --- |
| Lack of systematic standardized training programs and certification mechanisms | ○ | ○ | ○ | ○ | ○ |
| Shortage of senior pharmacists with combined obstetrics/gynecology and pediatrics expertise | ○ | ○ | ○ | ○ | ○ |
| Need to improve clinical practice skills (e.g., condition assessment, therapy adjustment recommendations) | ○ | ○ | ○ | ○ | ○ |
| Insufficient evidence-based pharmacy literacy and research capacity to support service innovation | ○ | ○ | ○ | ○ | ○ |
| Need to strengthen communication skills, patient education, and humanistic care | ○ | ○ | ○ | ○ | ○ |
| Difficulty attracting and retaining excellent specialty pharmacy talent | ○ | ○ | ○ | ○ | ○ |
| Insufficient continuing education opportunities and resources | ○ | ○ | ○ | ○ | ○ |

37. In terms of demonstrating service value and obtaining external recognition, what are the main challenges? (Rate importance: 1 = Not important at all; 2 = Slightly important; 3 = Moderately important; 4 = Very important; 5 = Extremely important) [Matrix] *

|  | 1 | 2 | 3 | 4 | 5 |
| --- | --- | --- | --- | --- | --- |
| Lack of unified national fee items and standards for women and children’s pharmacy services | ○ | ○ | ○ | ○ | ○ |
| Existing fee standards are too low to reflect the professional value of pharmacists’ work | ○ | ○ | ○ | ○ | ○ |
| Services not covered by medical insurance or have low reimbursement ratios | ○ | ○ | ○ | ○ | ○ |
| Low patient awareness and trust; weak willingness to pay | ○ | ○ | ○ | ○ | ○ |
| Clinicians’ limited recognition of value; low proactive referral | ○ | ○ | ○ | ○ | ○ |
| Lack of high-quality evidence demonstrating clinical and economic benefits | ○ | ○ | ○ | ○ | ○ |
| Insufficient media outreach and science communication; limited societal impact | ○ | ○ | ○ | ○ | ○ |

38. In terms of internal operations management and sustainable development, what are the main challenges? (Rate importance: 1 = Not important at all; 2 = Slightly important; 3 = Moderately important; 4 = Very important; 5 = Extremely important) [Matrix] *

|  | 1 | 2 | 3 | 4 | 5 |
| --- | --- | --- | --- | --- | --- |
| Heavy routine workload (dispensing, prescription review, etc.) limits time/energy for clinic services | ○ | ○ | ○ | ○ | ○ |
| Lack of scientific performance evaluation indicators and incentive mechanisms | ○ | ○ | ○ | ○ | ○ |
| Insufficient support for space, equipment, and IT investment | ○ | ○ | ○ | ○ | ○ |
| Lack of unified and detailed service specifications, SOPs, and quality standards | ○ | ○ | ○ | ○ | ○ |
| Incomplete IT systems (e.g., no dedicated modules; difficulty sharing data) | ○ | ○ | ○ | ○ | ○ |
| Suboptimal collaboration mechanisms (e.g., MDT, referral pathways) | ○ | ○ | ○ | ○ | ○ |
| Difficulty obtaining sustained research funding to support service innovation and optimization | ○ | ○ | ○ | ○ | ○ |

39. Besides the above, what other particularly difficult or representative problems have you encountered? [Fill-in] * *_________________________________*

# Part V. Future Development Planning & Industry Outlook

To promote high-quality establishment and sustainable development of your women-and-children specialty pharmacy outpatient clinics, which levels of support and actions are most urgently needed?

40. National/Government level (select 1–2 most important) [Multiple choice] *
□ Issue a dedicated development plan and supportive policies for women-and-children pharmacy
□ Establish a unified national catalog of service fee items and guidance prices
□ Include more mature service items into medical insurance payment coverage
□ Increase investment and support for training women-and-children specialty clinical pharmacists

41. Professional association/academic organization level (select 1–2 most important) [Multiple choice] *
□ Develop and promote service specifications, technical guidelines, or expert consensus
□ Establish training, assessment, and certification systems for specialty clinical pharmacists
□ Build platforms for academic exchange, experience sharing, and research collaboration
□ Organize quality evaluation and benchmarking programs

42. Hospital/Institution level (select 1–2 most important) [Multiple choice] *
□ Incorporate women-and-children pharmacy outpatient clinics into hospital strategy with sufficient staffing/resources/funding
□ Establish scientific performance evaluation and salary incentive mechanisms
□ Optimize internal referral processes and strengthen MDT collaboration
□ Strengthen IT construction and improve intelligent service capabilities

43. What is your attitude toward establishing a national/regional collaborative network for women-and-children pharmacy services (e.g., sharing difficult-case consultation resources, standardized training courses, clinical research data, distinctive service models, etc.)? [Single choice] *
○ Strongly supportive; urgent need; hope it can be realized
○ Supportive; certain need; hope it can be realized
○ Neutral / wait-and-see; depends on specific form and benefit
○ No obvious need or believe practical implementation is difficult
○ Not familiar with this

44. Does the hospital HIS have a dedicated pharmacy outpatient clinic module? ____ (Yes/No); Is AI-assisted decision-making used? ____ (Yes/No) [Fill-in] *

45. To promote standardized development of women-and-children specialty pharmacy outpatient clinics in China, what other key suggestions would you like to convey through this survey?

_________________________________

_________________________________
